# Parents’ Perspectives on Family Sexuality Communication from Middle School to High School

**DOI:** 10.3390/ijerph15010107

**Published:** 2018-01-10

**Authors:** Jennifer M. Grossman, Lisa J. Jenkins, Amanda M. Richer

**Affiliations:** Wellesley Centers for Women, Wellesley College, Wellesley, MA 02481, USA; ljenkins@wellesley.edu (L.J.J.); aricher@wellesley.edu (A.M.R.)

**Keywords:** teenage reproductive health, teen-parent relationships, family sexuality communication, adolescent development

## Abstract

Parents’ conversations with teens about sex and relationships can play a critical role in improving teenage reproductive health by reducing teens’ risky sexual behavior. However, little is known about how teen-parent communication changes from early to middle adolescence and how parents can tailor their communication to address their teens’ changing development and experiences during these periods. In this longitudinal qualitative study, U.S. parents (*N* = 23) participated in interviews when their teens were in early adolescence, then again when the teens were in middle adolescence. Participants were largely mothers and were from diverse racial/ethnic and educational backgrounds. Thematic analysis was used to assess continuity and change in parents’ perceptions of teen-parent communication. Findings showed that many parents adapted their conversations with their teens about sex and relationships as teens developed. Once teens had entered high school, more parents described feeling comfortable with their conversations. However, parents also more often reported that their teens responded negatively to the communication in high school than they had in middle school. These findings may help parents to anticipate their own as well as their teens’ responses to family conversations about sex at different developmental time points and to strategize how to effectively talk with their teens about sex and relationships to improve their teens’ overall reproductive health.

## 1. Introduction

Teens’ risky sexual behaviors, such as early sex, sex without a condom and having multiple partners have negative health outcomes [1]. Despite historically low birth rates for U.S. teens [2], rates continue to be higher than in other developed countries [3], and three out of four teen pregnancies in the U.S. are unintended [4]. Family conversations about sex and relationships provide one way to improve teen reproductive health by reducing teens’ sexual risk behavior [5,6]; however, teen-parent conversations about sex are only effective at reducing teens’ risk behavior when parents’ match their messages about sex with their teen’s developmental level and sexual experience [7,8]. Addressing teens’ developmental level is particularly important as teens transition from early to middle adolescence, as youth experience rapid developmental change in identity, sexuality and relationships during this time period [9]. Family sexuality communication can be further inhibited by many parents’ under-estimation of their teens’ sexual behavior [10], which may make it difficult for parents’ to effectively address teens’ developmental needs and could prevent teens from obtaining knowledge they need to reduce their sexual risk behavior [11]. Despite the need for developmentally appropriate family conversations about sex and the challenges parents face in achieving this goal, with few exceptions [12] little research assesses family sexuality communication over the transition from early (11–14) to middle adolescence (15–17).

Shifts in teens’ development from early to middle adolescence may complicate how parents talk with their teens about sex and how teens respond to these conversations. The transition from middle school to high school is a pivotal one for teen sexual activity. While 5–13% of adolescents report having had sex by 8th grade [13], 36% are sexually active by 10th grade [1]. By age 12, approximately 25% of teens report having had a romantic relationship, which doubles by age 15 [14]. To effectively navigate these changing roles and behaviors, many adolescents need information and support to negotiate social situations related to sex and relationships.

To support teens’ health, parents may face challenges related to noticing changes in their teens’ development and adapting their approaches to talking about sex and relationships to address them. Parents also need to be aware that teens’ growth from early to middle adolescence brings shifts in teen-parent relationships [15] such as growing independence and expanding social networks [16,17], which may lead to less openness or engagement with parents on the issues going on in their lives. Teens also show increasing influence of peer versus parent relationships from early to middle adolescence [18]. Therefore, teens’ questions and concerns related to sex and relationships, their willingness to talk with parents about sexual issues, and their ways of talking may change during this transition.

Many parents face their own challenges to talking effectively with their teens about sex and relationships. Barriers to parents’ talk with their teens about sex include parents’ lack of accurate information regarding sexual health, discomfort in talking about sex, and perceptions that their teens are not ready to talk about sex or engage in sexual activity [11,19]. Under-estimation of teens’ sexual activity is an area where some parents’ discomfort with their teens’ sexuality can intersect with teens’ development to impede effective teen-parent communication about sex. A large-scale study found that among sexually active teens, 55% of their parents incorrectly reported that their teens had not had sex, which may relate to social norms or parental beliefs against teen sex [10]. This has implications if parents’ messages about sexuality do not match teens’ needs and development. Inaccurate perceptions of teens’ sexual experience may prevent parents from providing key guidance to their teens on sexual issues [11]. For example, parents’ focus on delaying sex can be health-promoting for teens who have not had sex, but this message may miss the boat for sexually active teens, who might benefit more from information about protection methods to avoid pregnancy and sexually transmitted infections.

Parents’ and teens’ backgrounds also shape the content and style of sexuality communication, as well as its impact [20,21,22,23,24,25]. Much research investigates the role of teen and parent gender in family sexuality communication. The gender of both teens and parents can shape the frequency, content and impact of conversations. For example, parents are more likely to share messages with female than male teens that focus on abstinence and resisting a partner’s advances [20]. Other research shows that teen girls are more likely than boys to talk with family members about sex [21]. Findings are mixed as to whether family communication about sex is more likely to affect sexual behavior of male or female teens [5,21]. Few studies have explored change over time in parents’ sexuality communication with male and female teens. Family sexuality communication is also shaped by families’ racial and ethnic backgrounds [22,23], and research suggests that this communication can protect teens of different racial and ethnic groups from risky sexual behavior [24,25]. The current study explores family communication about sex and relationships within a racially and ethnically diverse sample, but a comparison of racial/ethnic groups is beyond the scope of this paper.

While few studies assess continuity and change in parent-teen talk about sex and relationships, one quantitative study that followed adolescents over a 12-month period found that once teens became sexually active, parents’ messages about sex focused more on issues such as how to choose a birth control method and recognize symptoms of sexually transmitted infections [12]. At the same time, cross-sectional research suggests that once teens become sexually active, they may be less likely to talk with parents about their sexual thoughts and experiences due to fear that their parents might judge them or worry about their sexual behavior [26,27] or concern that their parents may try to control their developing sexuality [28]. In contrast, a 4-year longitudinal study of late adolescents found that participants felt that both themselves and their parents became more comfortable and open in talking about sex over the course of college as they developed [29], consistent with growing mutuality in teen-parent relationships during emerging adulthood [30]. These studies suggest the importance of adolescents’ development when understanding how parents and teens talk about sexuality. While the periods of early and middle adolescence include significant change in identity, sexuality and relationships, there is a gap in research assessing change in teen-parent talk about sex during this developmental transition.

The lack of studies on change over time in teen-parent sexuality communication and the critical importance of developmentally appropriate parental conversations with teens show the need for longitudinal research in this area. The current study provides a unique longitudinal examination of parents’ perceptions of continuity and change in teen-parent communication from middle school to high school. The knowledge gained from this study will guide our understanding of how parents do or do not adapt their approaches to sexuality communication to teens’ changing development and sexuality. It also explores the role of teen gender in shaping parents’ approaches to talk with teens about sex and relationships and the content of these conversations.

## 2. Materials and Methods

### 2.1. Recruitment and Participants

Our interview sample consisted of parents of adolescents from three schools that participated in an evaluation of *Get Real: Comprehensive Sex Education That Works*, a three-year program developed by Planned Parenthood League of Massachusetts. *Get Real* is a comprehensive sexuality communication program which emphasizes delaying sex while providing medically accurate information about protection. It identifies parents as the primary sexuality educators of their children and includes supports for teens and their family members to talk with each other about sexuality and relationships. *Get Real* has been shown to be effective in delaying sex for middle school students [5]. Researchers interviewed participating parents twice: once when teens were in seventh grade and again when teens were in tenth grade. Schools were selected for the interview study because their student populations were demographically representative of the larger evaluation, which included 24 schools (see [5] for a further description of the evaluation study). Active consent was required for interview participation. A letter was distributed to inform parents about the interview study and to invite them to participate. Each school decided how consent forms were distributed (see Grossman and colleagues for details [31]). Approximately 177 parents/guardians were invited to participate in interviews, and 38 consent forms were returned, with four parents not consenting to participate. All parents who provided consent were contacted for an interview; 94% of those (32 parents/guardians) completed interviews (Time 1). Only one parent in each family was interviewed. Given the focus of the current study on teen-parent communication, three participating guardians (great aunt, grandmother, older sister) were excluded from this analysis. Each parent completed a demographic questionnaire.

Participating parents were invited to participate in follow–up interviews three years later, when parents had high school-aged teens (Time 2). Parents were contacted by e-mail and phone and asked to complete active consent forms for the follow-up study. Of the 29 original parent participants, 23 agreed to participate and completed interviews at Time 2, representing 79% of the original parent sample; 6 families were unreachable. For both Time 1 and Time 2, interviews were conducted over the phone primarily in English (2 were conducted in Spanish), took 30–45 min and were later transcribed and translated as needed. Parents were each given $25 in appreciation of their participation. Each participant was asked to create a code name to protect confidentiality; those pseudonyms are used here. All participating parents were provided with a resource list with contact information for organizations supporting youth and family social, emotional, and sexual health. At each time point, human subjects approval was granted from The Institutional Review Board at Wellesley College to conduct this work (January 2011 and December 2013). Only parents who participated in both Time 1 and Time 2 interviews were included in this paper. Within our sample of 23 parents, 20 parents were mothers and three parents were fathers. Fifty percent of participants self-identified as Black/African American, 13% as Hispanic/Latino, and 37% as White. Thirty-eight percent of participants reported completing high school or less education, 25% reported some college education, 29% reported college or additional education, and 8% did not respond to this question. Thirty-seven percent of participants reported single-parent status, 46% reported living in two-parent families, and 17% reported living with a parent as well as another adult family member. Demographics of participants who participated in both Time 1 and Time 2 interviews were similar to the 29 participants who participated in Time 1 interviews [31].

### 2.2. Interview Protocol

Prior to interviews, participants were reminded of the purpose of the study and reassured that it would be normal to feel a bit embarrassed or uncomfortable, and that they could choose not to answer any questions. Interview questions addressed parents’ communication with their adolescent children about sex and relationships at each time point. Specifically, we asked parents about the content of their communication with their teens, their comfort with this communication, and their understanding and experiences of talking with their teens about sexual issues.

### 2.3. Data Analysis

We used thematic analysis to systematically identify themes in the interview data [32]. The first and second authors closely read the transcribed interviews and separately identified overarching themes in parents’ Time 1 and Time 2 data that represented patterns in parents’ perceptions and experiences of sexuality communication with their teens. The authors then coded interview data, reviewed and revised themes based on how they fit with coded data, defined and named them and developed sub-themes [33]. The themes were then compared across parents’ Time 1 and Time 2 interviews to explore continuity and change in parents’ perceptions of sexuality communication when teens were in middle school and high school. The themes were not mutually exclusive, in that one participant’s responses could generate more than one code. The 1st and 3rd authors conducted reliability checks [34]. The intercoder reliability of 94% represented a high level agreement between the two coders. NVivo 10.0 (QSR International, Melbourne, Australia) [35] was used to facilitate coding.

## 3. Results

Six overarching themes emerged from parent interviews. The first three themes reflect parents’ experiences of and approaches to communication with their teens about sex and relationships: Reasons for sexuality communication, Comfort (or discomfort) talking about sex, and Perceptions of teens’ experiences of sexuality communication. The remaining themes focus on whether and how parents talk with their teens about specific content areas, namely Talk about dating & relationships, Talk about readiness for sex, and Talk about sexual risk and protection. See Table 1 for a comparison of themes for Time 1 and Time 2 interviews.

### 3.1. Reasons for Sexuality Communication

In the *Reasons for sexuality communication* theme, parents talked about the reasons they talked more or talked less with their teens about sex and relationships with sub-themes of *Why talk more* and *Why talk less*. At Time 1, 78% (18/23) of parents described their reasoning for why they talked with their teens about sex, whereas 65% (15/23) of parents at Time 2 did so. Parents of male and female teens reported similar rates of why they talk at Time 1 and Time 2. Parents at both time points described a focus on protecting teens from future risk. At Time 1, Jada explained why she talked with her teens about sex, “I wanted to empower my kids by giving them the tools, the language, the understanding, because no matter how much you prepare your kids for situations that you would rather they not have an experience with, sometimes it doesn’t pan out”. Judy shared, “I would much rather [my children] ask me questions than just be blindsided by reality”. Parents at Time 2 shared similar responses. Julia explained, “They’re going to face all those things in life, so you have to talk about everything. I don’t want them to be in pain and I want them to stay to school for their education and find someone to marry, to continue their life and have a family”. Derrick explained, “I constantly always bring it [sex] up, you know, just to make sure she don’t do nothing that I consider stupid”.

Differences in reasons parents gave for talking with their teens about sex at Time 1 compared to Time 2 emerged. At Time 1, parents talked about the importance of teens getting accurate information about sexual issues and described teens initiating conversations about sex, whereas at Time 2, parents described talk about sex because of teens’ interest or involvement in romantic and/or sexual relationships. For example, at Time 1 Maria explained, “I want my son to learn from me before he learns from one of his friends in school”. Ellen explained why she talks with her daughter about sex, “so she’s educated and she has the right information, and not the wrong information”. Parents at Time 1 also described their teens’ questions as a motivation for talking with them about sex. For example, Kevi said, “He [my son] actually brought it up. He had a few questions about sex and he wasn’t sure if what he had heard was true”. Another parent (Barbara) shared, “[My son] asked me the names of the parts of the penis and the names of the parts of the vagina, and then it was time to tell him how things really are.” At Time 2, parents identified teens’ interest or involvement in relationships as a reason for talking with them about sex. Alex explained her increased communication with her son about sex, “because he has a girlfriend, so we talk a lot more now.” Susan shared, “I thought it was not necessary too much before because she wasn’t really hooking up with people. But now she’s more social and she’s going to be more adult now. She wants to have a boyfriend, that’s when I know it’s probably the time”.

A similar number of parents at Time 1 (39%, 9/23) and Time 2 (43%, 10/23) described why they rarely or never talked with their teens about sex. Parents at both time points described not talking with their teens because they perceived them as not being ready or mature enough to talk about sexual issues or due to perceptions that the teens were not interested or engaged in dating. At Time 1, parents were more likely to describe reasons why they did not talk with female (55%) than male teens (17%) about sex, but differences were less evident at Time 2 (females = 36%, males = 50%). At Time 1, Carmel explained, “She’s too young right now. I don’t want to put ideas in her head”. Similarly, Rose shared, “We didn’t go over details because she’s just waiting to get her period, so she’s kind of like a little girl still”. At Time 2, Jasmine described why she doesn’t talk with her daughter about sex, “I don’t think she’s ready. When she’s ready, she’ll come to me,” Another parent at Time 2 (Marcia) explained why she rarely talks with her son about sex, “[Dating] is not one of his interests right now…so we don’t have to talk about it”.

### 3.2. Comfort Talking about Sex

The second theme relates to parents’ Comfort (or discomfort) in talking with teens about sex with sub-themes of *Comfort* and *Discomfort*. More parents expressed comfort with sexuality communication at Time 2 (96%, 22/23) than at Time 1 (65%, 15/23). At Time 1, *Comfort talking about sex* was described more frequently by parents of female (82%) than male teens (50%), but differences were not evident at Time 2 (females = 100%, males = 92%). At Time 1, Cordelia shared, “I don’t have any trouble talking to my kids about sex”, while Julia stated, “I’m pretty comfortable, it’s just that sometimes I don’t know exactly what to say”. At Time 2, many parents discussed ways in which their comfort talking about sex had grown since their children were in middle school, often related to perceived changes in their teens’ increased age and maturity. For example, Jenny described talking with her daughter about sex, “I’m really comfortable. I feel she’s older so she’s able to understand a little bit more than she did in middle school”. Barbara described what it’s like to talk with her son in high school, “I feel more comfortable because [my son] is more of an adult. And he knows more about sex and about relationships”. A third parent, Maria, described her growing ease in talking with her son about sex, “I think the maturity. He’s bigger and it’s he’s somewhat more serious now. So we have good communication”.

Several parents described discomfort in talking with their teens about sex at Time 1, (39%, 9/23), whereas only one parent described discomfort at Time 2 (4%, 1/23). At Time 1, parents more often described discomfort in talking with male (50%) than female teens (27%), but these differences were not evident at Time 2 (males = 8%, females = 0%). Some female parents at Time 1 described difficulties in talking with their sons about sexual issues. One mother, Alex, explained, “It’s just the comfort of him being the male child. It feels weird for me to sit with my son and have these conversations”. Some parents at Time 1 discussed the importance of talking with their children about sex despite their discomfort with these conversations. Jada explained, “I’m still somewhat uncomfortable…to me it’s more the awkwardness of finding the right words at the right time. But, you know, the sigh of relief is, no matter how uncomfortable I am, I’m willing to do it”. A father (Ryan) explained, “[My daughter] will just randomly ask like how old was I, or what age you think is too young [to have sex] and it kind of throws me off. But I do appreciate that it’s open enough for her to ask me something like that”.

### 3.3. Perceptions of Teens’ Experiences of Sexuality Communication

The third theme, *Perceptions of teens’ experiences of sexuality communication*, focuses on how parents perceive teens’ behavioral and emotional responses to talking with a parent about sex with sub-themes of *Positive engagement* and *Negative engagement*. At both time points, parents described variation in teens’ responses to talking with parents about sex, ranging from comfort and engagement to discomfort and avoidance. At Time 1 (70%, 16/23) and Time 2 (65%, 15/23) more than half of parents described ways their teens showed positive engagement in talking with them about sex. There were no evident differences in parents’ reports of positive engagement of male or female teens at either Time 1 or Time 2. At Time 1, parents often described teens asking them questions, such as Ellen, whose daughter asked, “Mom, what’s a dental dam?” Kevi described her son’s communication about sex at Time 1, “He is continually asking questions. He’s very open.” Alex described her son’s engagement when they talked about sexually transmitted infections, “I was like, ‘That’s the way that you can catch something that you can never, ever, ever get rid of because there’s no medicine for it.’ He was like ‘For real?’ I was like, ‘Yeah, for real.’” At Time 2, parents described their perceptions of teens’ engagement primarily through showing they were listening and seeming open and comfortable with conversations. For example, Jada described her son’s response to her communication, “He kind of listens and just doesn’t say anything. It’s like he’s putting it in his pocket somewhere, and maybe he’ll use it someday.” Marie talked about her children’s response to her talk about sex, “They’re so used to it that I think talking to me is just like, ‘Oh, I’m talking to one of my friends’”.

More parents described negative teen responses to talk about sex at Time 2 (65%, 15/23) than Time 1 (39%, 9/23), which include avoidance or negative reactions to parent talk about sex, such as appearing embarrassed or uncomfortable and saying they already knew the information parents provided. There were no evident differences in parents’ reports of negative engagement of male or female teens at either time point. At Time 1, Jean described her son’s response to her and her husband raising the topic of sex “He just kind of looked at us with a blank look like, ‘Shut up. I don’t want to hear it.’” Parents described similar struggles at Time 2, and some pointed out an increased level of discomfort with talking about sex as teens got older. For example, Kevi shared about her son, “he just seemed more open when he was younger than he is now. I think he gets more embarrassed.” Lynn stated, “I talk all the time [about sex] and he rolls his eyes.” Jasmine described her daughter’s response to her raising sexual topics, “She’ll say, ‘Ma, I already know. I already know’”.

### 3.4. Talk about Dating and Relationships

The next three themes described the content of parents’ conversations with their teens related to sex and relationships. The first content theme addresses how parents and teens *Talk about dating and relationships*. Almost all participating parents at Time 1 (91%, 21/23) and Time 2 (96%, 22/23) reported talking with their teens about this topic. However, the content of these conversations differed for Time 1 and Time 2. At Time 1, the most common focus of conversation described by parents was rules for teen dating and relationships, while at Time 2 conversations focused more on teens’ interest or involvement in dating and how to have a healthy relationship. There were no evident differences in parents’ frequency of talk with male or female teens about dating and relationships at either Time 1 or Time 2. At Time 1, Jean explained how she talks with her son about dating: “He’s girl-crazy right now and my husband and I are very adamant about there’s no need for thirteen year olds to have relationships. ‘It’s normal to have those feelings. It’s the self-control and acting on them that you have to really remember what you were taught and realize that that’s not okay, because it can go further.’” Cordelia shared a similar message with her daughter about dating at Time 1: “I think it’s premature now, they’re 13. I tell them I don’t know the number because I don’t know how mature they’re going to be at age X. So I’d rather not put an age out there until I see them at that age.” Other parents at Time 1 described giving their children permission to do some level of dating, but set limits on what activities were permitted. For example, Tiffany explained her message to her son, “If you see a girl that you particularly like, okay fine. You can go to McDonalds, you can go to the movies. But you know, that’s pretty much it”.

In contrast, at Time 2 many parents described conversations about teens’ interest or involvement in relationships and how to have a healthy relationship as opposed to rules about dating. Parents also reported that some of these conversations were initiated by teens. For example, Jasmine shared, “I can say she’s a teenager right now and she tells me things like people like her or this boy likes her.” Cordelia reported, “We’re constantly having discussions about their feelings—they find this one attractive, they find that one attractive.” Parent-teen conversations about healthy relationships often included feedback to teens on how to be in a healthy relationship. For example, Daisy shared the messages she passes on to her son, “I gave him advice about dating, that he’s young and he’s going to meet a lot of girls but don’t get into a really serious relationship. Just get to know the person.” Kevi described a conversation with her son, “I always tell him how to have respect for his girlfriend and it will come back to him as well, and never to be in a bad relationship”.

### 3.5. Talk about Readiness for Sex

The second content theme, *Talk about readiness for sex*, includes sub-themes of *Concrete reasons to delay sex* and *Emotional and relational reasons to delay sex*. Concrete reasons for delay focus on specific time points or life events such as finishing school, getting married, or reaching a certain age (e.g., 18, 21) before having sex. At Time 1, 61% (14/23) of parents described talking with their teens about concrete reasons to delay sex, whereas only 39% (9/23) of parents at Time 2 reported talking about this issue. At both Time 1 (males = 75%, females = 46%) and Time 2 (males = 50%, females = 27%), parents more often described talking with male than female teens about concrete reasons to delay sex. At Time 1, delaying sex until finishing school or getting married were the two primary points of discussion. For example, Daisy shared, “Before you make any decisions about having sex, I really want you to have your education finished, go to college.” Jean stated, “For us sex comes after marriage, which is a covenant with God. So there’s nothing outside of marriage that makes sex okay.” Some parents raised both these issues, such as Barbara, “I told him he has to wait until getting married to start a relationship with a girl. I think education comes first.” As with Time 1, at Time 2 some parents described conversations about finishing school and waiting until a teen was older before having sex. For example, Ellen stated, “You’re too young; you need to live life. Get an education; go to college. Leave the state; see the world.” Jada described a conversation with her children about when they’re old enough to have sex, “I tell them ‘I hope you’re at least twenty-one before you do.’” In contrast, delaying sex until marriage was rarely discussed at Time 2.

The second sub-theme for *Talk about readiness for sex* addressed emotional or relational reasons for delay focused on teens’ emotional maturity and being in a close, committed relationship before having sex. Half of parents at both Time 1 (48%, 11/23) and Time 2 (48%, 11/23) described talking with their teens about emotional or relational reasons for delay. At Time 1 parents more often described talking with male (58%) than female teens (36%) about emotional or relational reasons to delay sex, but this was not evident at Time 2 (males = 50%, females = 46%). Parents described similar content of conversations at both time points. For example, at Time 1 Lynn shared, “I have stressed to both of my sons the importance of being really ready and really in love before having sex. That it’s not something to be casual about.” Similarly, Rose explained how she talks with her daughter about sex, “I want her to be in love, not to feel cheap or any of that type of thing. I want her to be really ready.” At Time 2, Marie shared her advice to her daughter, “I feel like if you have self-respect and maturity, that’s going to really help you decide when the time’s right for you. If you love yourself enough, you’re not going to need to have sex with someone to define yourself.” Cordelia described her advice to her daughter about sex, “focus on being in a loving relationship with somebody that doesn’t hurt you, that you’re committed to and he’s committed to you”.

### 3.6. Talk about Sexual Risk and Protection

The third content theme addresses how parents and teens *Talk about sexual risk and protection*. Most parents at Time 1 (96%; 22/23) and all parents at Time 2 (100%; 23/23) reported talking with their teens about this topic often emphasizing teen pregnancy and parenting, sexually transmitted infections (STIs), and protection methods. There were no evident differences in parents’ frequency of talk with male or female teens about sexual risk and protection at either Time 1 or Time 2.

Parents at both time points described talking with teens about teen pregnancy and parenting, focusing on the hardships of teen parenthood. Some parents talked with their children about the challenges that they or other family members faced as teen parents. At Time 1, Ellen described talking with her daughter about her own mother’s struggles as a teen parent, “My mother had me at 15. We talk about that a lot, and I tell her how my mother’s life was not easy. She had a really, really hard time for a lot of years.” At Time 2, Marcia described how she talks with her son about her own experiences, “I just always tell him, ‘Don’t be like me, because life is hard.’ You know? And probably if I had waited until I was older, then I would have more education and stuff like that and then now it wouldn’t be such struggle.” At Time 2, many parents elaborated on the importance of taking responsibility for teen parenting roles. For example, Carmel shared, “I say, ‘You’re going to take care of the child yourself. You and your partner—wherever he is—are going to take care of it [your child] yourself. I have raised mine, so that’s your responsibility.” Similarly, Julia explained what she said to her son about getting someone pregnant, “If you have a lady who gets pregnant, you know what’s going to happen? In tenth grade? You have to leave school. You have to work at McDonalds, because I’m not going to take care of more people than what I’m taking care of now”.

Parents at both time points talked about risks of sexually transmitted infections (STIs). At Time 1, Tiffany warned her son about STIs, “I try to let that be known that you hear about the girls going around doing blowjobs and stuff, but you can still pick up STDs through blowjobs. So I know it seems so open, but I really feel like they need to know this.” Marie shared, “I tell my children, ‘It’s so much more than you just laying down and having sex.’ there could be unwanted pregnancies, it could be a death sentence, or an STD.” Similarly, at Time 2, Rose explained to her daughter, “You just have to be careful of sex with different people and people carry diseases and that type of thing”.

Parents at both time points described talk with their teens about the importance of using protection methods, particularly condoms, to avoid pregnancy and STIs. Parents’ description of conversations with their teens at both time points included general directives to use protection and specific plans to access condoms. At Time 1, Daisy described her comments to her son, “You’ve got to be careful. There’s a lot of diseases. You’ve got to wear a condom.’ Tiffany explained, “I can tell him ‘no’ until I’m blue in the face, but you know, he gets that urge and he still wants to try it, he’s going to try it regardless. We try to let him know when that time comes, just come to us and his dad will give him the condoms or he gets some sort of access to condoms.” Parents at Time 2 passed on similar messages. For example, Carmel recalled a conversation with her daughter, “All I say is: ‘If you do get ready [to have sex], make sure your partner has condoms, and if they don’t, you’re not having any. You might get anything: gonorrhea, syphilis, anything that it has no cure for. Then, what you going to do?’” Judy explained how she talked about protection with her son, “When he’s ready to take that step [having sex], he made that vow that he’s going to come to me and I told him it may seem weird to some people, but I’ll be the first one to go out and buy him condoms.” In contrast, a small minority of parents expressed concerns that providing condoms might encourage their teens to have sex. For example, at Time 1, Marcia recalled a conversation with her son, “He tells me that he has it [a condom] in his locker at school. I said, ‘Uh-uh. Don’t keep it there at school. Bring it home. When you have it in school, you might get ideas’”.

## 4. Discussion

This study is one of the few to longitudinally investigate teen-parent communication about sex and relationships and the only one to our knowledge that explores continuity and change in parents’ perspectives of sexuality communication. It provides an opportunity to explore how parents navigate talking with their teens about sex and relationships among a diverse group of parents in the context of change in teens’ development as well as teen-parent relationships from early to middle adolescence. This study explores how parents approach conversations with their teens about sex and relationships, what they talk about, how they perceive their teens’ reactions to these conversations and finally, the role of teen gender in this communication.

This study’s findings show that many parents adapted their conversations with their teens about sex and relationships as teens developed. For example, parents at Time 2 noted that teens’ expressed interest in dating motivated parents to talk with their teens about sex and relationships, which was not evident at Time 1. Similarly, parents’ approaches to talking with teens about dating shifted from rules and restrictions at Time 1 to discussions of relationship experiences and how to have healthy relationships at Time 2, which suggests an awareness of teens’ changing developmental needs from middle school to high school. Further, more parents at Time 1 than Time 2 talked with their teens about concrete landmarks for delaying sex (e.g., finishing school, turning 18, getting married). This may indicate parents’ awareness of teens’ growing autonomy in making their own decisions about sexual behavior or growing acceptance of sex as a normative part of teens’ experience as they get older. With few exceptions, parents in this sample showed shifting approaches to talk with teens from middle school to high school which suggest parents’ responsiveness to teens’ development. Despite parents’ shifts over time toward more positive approaches to teens’ relationships, their conversations with teens about sex remained focused on its negative consequences, rather than addressing any potential benefits.

A complexity with parents’ responsiveness to perceived changes in teens’ development is that parents may not always accurately perceive teens’ interest in or readiness for relationships and sex. For example, some parents described delaying talk with their teens about sex due to a belief that their child was not interested in dating. Whereas some parents may accurately perceive their teens’ lack of interest in dating or relationships, other parents may under-estimate teens’ involvement in relationships and sexual activity [10]. A longitudinal study of the timing of parent-teen conversation about sex found that over 40% of teens have sex before having conversations with parents about protection methods or sexually transmitted infections [12]. Waiting until teens themselves raise questions about sex or until teens are perceived as “ready” may prevent parents from providing key guidance to their teens on sexual issues [11,36].

In contrast to changing parental approaches to conversations with teens about dating and sex, communication about teen pregnancy, STIs, and protection methods showed little change in frequency or content over time. The high level of parent communication about these topics indicates that parents see these issues as educational for their teens. However, the lack of change in content from early to middle adolescence suggests that parents may not yet perceive their teens as immediately at risk for pregnancy or STIs even in the high school sample. Further interviews with parents of teens who are later in high school or post-high school could inform whether parents approach these conversations differently with a late adolescent/emerging adult sample.

The findings from this paper also show a complex interplay between parents’ and teens’ shifting responses to talk about sex over time. Many parents described their own growing comfort in talking with teens about sex and relationships as teens got older and they described high school-aged teens as more able to understand and discuss sexual issues. However, more parents at Time 2 than Time 1 noticed teens’ negative responses to parents’ comments about sex or relationships, consistent with prior research identifying teens’ reluctance to talk with parents about sex when adolescents become sexually active [26,27]. These findings may help to explain challenges parents and teens face in talking with each other about sex and relationships, particularly later in adolescence. Parents’ level of comfort (or discomfort) and teens’ engagement (or lack of) with talk about sex may fluctuate over time and could impede effective communication. However, even when parents expressed discomfort with talking with teens about sex and relationships, they often described their commitment to talking despite this discomfort. Similarly, a study of mothers’ communication with teens about sex found that “mothers push through their discomfort…because they believe the stakes are too high” (p. 317) [36]. Elliot suggests that by anticipating and normalizing teens’ resistance to talking with parents about sex, parents can more effectively overcome these barriers.

Teen gender also shaped parent comfort and engagement with sexuality communication as well as its content, particularly at Time 1. Parents were more likely to describe reasons for not talking with their daughters than sons about sex and relationships, often referring to their daughters’ lack of readiness to discuss sexual issues and perceptions of them as “little girls”. This may reflect parents’ reluctance to see their teen daughters as sexual beings, which has been suggested in prior work on parents’ attitudes toward teen girls’ sexuality [20]. This may also be reflected in parents’ lower frequency of reported talk with their daughters than sons at Time 1 about delaying sex. While research suggests that parents are more likely to talk with daughters than sons about abstinence [20], some parents may postpone these conversations with daughters until middle adolescence as they don’t yet perceive early adolescent girls as ready to engage with sexual issues. Interestingly, despite parents’ lower frequency of talk with their daughters than sons, they describe more comfort in talking about sex with daughters than sons. Some mothers specifically discussed discomfort in talking with sons because of their gender. This complements prior findings that teens can be more comfortable talking with same-sex family members about sex and relationships [37].

Parents’ and teens’ struggles to talk with one another about sex and relationships and the role of gender in these interactions suggest the importance of exploring other teen resources for conversations about sex, in addition to parents. Research shows that over half of teens talk with extended family, such as stepmothers, grandparents, uncles, cousins, and siblings about sex [38,39]. This is particularly relevant for Black and Latino teens, who more often have non-parental family members involved in their upbringing [40,41]. Teens also talk with peers about sex and relationships, although findings suggest that these conversations are not always health-promoting [42,43]. Further research is needed to understand the role non-parental relationships play in talking with teens about sex and relationships and whether and how this communication can support teens’ health.

In the current sample of highly engaged parents, parents were likely to talk with teens despite their own and their teens’ discomfort in addressing sexual issues. However, this persistence may not be typical among a more representative parent sample. Families’ participation in a school-based sex education program may also have increased their engagement with family sexuality communication. Finding ways to include parents who are less engaged in family sexuality communication in research studies would help to assess to what extent this study’s findings are generalizable to a broader population. In addition, the sample size is small and results should therefore be seen as preliminary. Future research would also benefit from a matched, longitudinal design that includes perspectives on family sexuality communication from both parents and teens when teens are in early and middle adolescence, which would provide a comparison to how parents may perceive (or misperceive) their teens’ responses to conversations about sex and relationships. While analysis by racial and ethnic group is beyond the scope of this study, their role in shaping family conversations about sex and relationships [22,23] suggests that future studies would benefit from exploring similarities and differences across racial and ethnic groups. Additionally, interactions of family sexuality communication and social class would help to understand variation across family contexts. Finally, it is important to include more fathers in research on family sexuality communication, a group that is under-represented in this area of study [24]. Inclusion of fathers could provide insight into how their perspectives on talking with teens about sex and relationships over time are similar or different than those of mothers, who represented the majority of the current sample.

These findings provide evidence of parents’ responsiveness to their teens’ age, development and experiences in their conversations with teens about sex and relationships. However, combined with prior research on parents’ under-reporting of teens’ sexual behavior [11], this study’s findings raise questions about whether some parents may wait too long to begin talking with their teens about sex and relationships, which could impact teen’s sexual health by resulting in unwanted teen pregnancies or sexually transmitted infections. This study also reiterates the challenges of talking with teens about issues that often make both teens and their parents uncomfortable and highlights developmental shifts that may make parent-teen conversations easier or more difficult at different stages of adolescent development. To counter teen resistance, parents may need to make extra efforts to engage teens in open and non-judgmental ways. These findings may help parents to anticipate their own as well as their teens’ responses to family conversations about sex at different developmental time points, normalizing potential negative responses and helping parents to strategize how to overcome both their own and teens’ discomfort to achieve effective communication about sex and relationships.

## 5. Conclusions

Family sexuality communication can provide a protective tool to improve public health outcomes by reducing teen pregnancy and sexually transmitted infections. However, in order to talk effectively with teens about sex and relationships, parents need to talk with teens in ways that developmentally fit their age and experiences. Talking with teens about sex is a challenge for many parents [11,19] and the added complexity of matching it with teens’ development is not an easy task. Health education programs that include outreach to parents can help to increase parents’ skills and comfort in talking with teens about sex and relationships [44,45]. While some school and community-based programs provide guidance for parents, more resources/supports are needed to provide parents with the tools they need to support teens’ reproductive health and reduce these public health concerns. Since parents are one key resource for teens’ sexuality communication [46], helping them to talk in open and developmentally appropriate ways with their teens and manage potential challenges of their own and their teens’ discomfort in talking about sex and relationships can make a difference in supporting teens’ reproductive health.

## Figures and Tables

**Table 1:** Parents’ perceptions of parent-teen sexuality communication at Time 1 and Time 2 (*N* = 23).

Theme	Time 1	Time 2
Reasons for sexuality communication		
Why talk more	18 (78%)	15 (65%)
Why talk less	9 (39%)	10 (43%)
Comfort talking about sex		
Comfort	15 (65%)	22 (96%)
Discomfort	9 (39%)	1 (4%)
Perceptions of teens’ experiences of sexuality communication		
Positive Engagement	16 (70%)	15 (65%)
Negative Engagement	9 (39%)	15 (65%)
Talk about dating and relationships	21 (91%)	22 (96%)
Talk about readiness for sex		
Concrete reasons to delay sex	14 (61%)	9 (39%)
Emotional & relational reasons to delay sex	11 (48%)	11 (48%)
Talk about sexual risk & protection	22 (96%)	23 (100%)
